# Comprehensive prognostic gene identification and functional characterization of GRAMD1A in Wilms tumor: development of risk prediction models and therapeutic implications

**DOI:** 10.3389/fonc.2024.1501718

**Published:** 2024-11-26

**Authors:** Qiang Zeng, Junfeng Tao, Lilu Qin, Yong Zeng, Zhong Liu, Mingxian Xu, Linshan Zeng

**Affiliations:** ^1^ Department of Pediatric Surgery, Jiangxi Maternal and Child Health Hospital, Jiangxi Children’s Medical Center, Nanchang, Jiangxi, China; ^2^ Department of Pediatric Surgery, The First Affiliated Hospital of Gannan Medical University, Ganzhou, Jiangxi, China; ^3^ Department of Musculoskeletal Oncology, The First Affiliated Hospital, Sun Yat-sen University, Guangzhou, Guangdong, China

**Keywords:** Wilms tumor, GRAMD1A, prognostic markers, LASSO regression, cell cycle

## Abstract

**Background:**

Wilms tumor (WT) is the most common pediatric kidney cancer, with survival rates exceeding 90% in localized cases. However, advanced or recurrent WT remains difficult to treat due to poor prognosis and limited knowledge of its molecular mechanisms. Gene expression profiling has shown promise in identifying prognostic markers and therapeutic targets. This study aimed to identify key prognostic genes and pathways in WT, construct risk prediction models, and validate their role in tumor progression.

**Methods:**

RNA sequencing and clinical data from 136 WT patients were obtained from the TARGET database. Differential gene expression analysis was conducted using GEO datasets GSE11024 and GSE66405 to compare WT and normal kidney tissues. Identified differentially expressed genes (DEGs) underwent Gene Ontology (GO) and KEGG pathway enrichment analysis to explore biological functions and pathways associated with WT progression. Univariate Cox regression was used to assess the association between DEGs and overall survival (OS) and progression-free survival (PFS). LASSO regression models were developed for risk stratification, and model accuracy was evaluated using time-dependent ROC curves. External validation confirmed key hub genes, while functional assays in WT cell lines (WiT-49) assessed the role of GRAMD1A in tumor behavior.

**Results:**

A total of 3,395 DEGs were identified, with 1,564 upregulated and 1,831 downregulated genes. Enrichment analyses revealed significant pathways involved in cell cycle regulation and metabolic reprogramming. Six key genes (GRAMD1A, PLXNA3, SPR, EBAG9, RBM47, and RIDA) were associated with both OS and PFS. LASSO models demonstrated strong predictive performance, with GRAMD1A identified as a major risk factor. External validation confirmed differential expression, and functional assays showed that GRAMD1A silencing significantly inhibited WT cell viability, proliferation, migration, and invasion.

**Conclusions:**

This study identifies novel prognostic genes and potential therapeutic targets in WT. GRAMD1A, SPR, EBAG9, RBM47, and RIDA play critical roles in WT progression, with GRAMD1A as a key oncogenic factor, offering potential for risk stratification and future therapeutic intervention.

## Introduction

Wilms tumor (WT) is the most prevalent renal malignancy in infants and young children (8, 9). WT is closely linked to early nephrogenesis, exhibiting morphological and genetic similarities with the early stages of kidney development (10–12). Standard treatment regimens primarily involve a multimodal approach comprising surgery, chemotherapy, and radiotherapy. Despite significant reductions in mortality through advancements in these therapies, the issues of tumor recurrence and treatment-related toxicity persist, highlighting the urgent need for more individualized therapeutic strategies (13, 14). While nephrectomy remains a widely accepted and effective treatment, it presents significant challenges in pediatric patients, particularly in those undergoing active growth or with bilateral involvement (15–18).

Clonal evolution in tumor cells is shaped by both morphological and genetic progression. Numerous copy number alterations (CNAs) serve as prognostic biomarkers for patient stratification. For instance, the WT1 tumor suppressor gene, which is critical in kidney development, has been shown to play a pivotal role in Wilms tumor (WT) pathogenesis. Mutations in WT1 are associated with abnormal tumor formation and developmental defects (19–21). Thus, optimizing therapeutic strategies based on clinical and biological risk factors is key to advancing personalized treatment and improving patient outcomes (5, 11, 22). A deeper understanding of tumor biology is essential for elucidating the genetic basis of WT and facilitating the development of personalized therapeutic approaches. Although significant progress has been made, the molecular mechanisms driving WT development, and the identification of potential therapeutic targets remain incompletely understood. In this study, we aim to identify potential prognostic and therapeutic targets, as well as the underlying molecular mechanisms of WT, by leveraging data from the TARGET and GEO databases. Additionally, we seek to construct a reliable clinical prognostic model based on key genes, offering promising therapeutic avenues for WT patients.

## Methods

### Dataset retrieval and preprocessing

**Figure 1:**
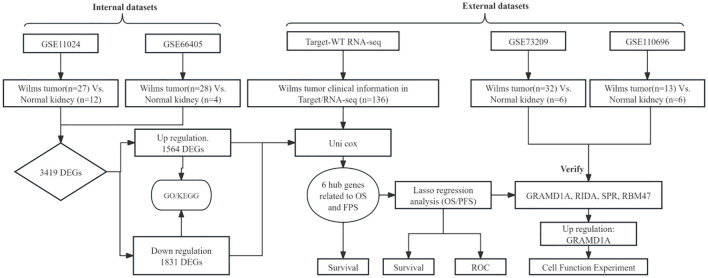
Schematic overview of the analytical workflow employed in this study.

The overall analytical process is depicted in **Figure 1**. RNA-seq data were sourced from the TARGET database, encompassing clinical data from 136 Wilms tumor (WT) cases. In addition, the GSE11024 (1), GSE66405 (2),GSE73209 (3), and GSE110696 (4) datasets were retrieved from the NCBI GEO database (http://www.ncbi.nih.gov/geo). **Table 1** outlines the specific applications of these datasets in the study.

**Table 1:** Overview of principal attributes of the datasets employed.

Dataset	Country	Platforms	No. of samples	Usage here
Target-WT	USA	RNA seq	Wilms tumor (n=136)	Development and Validation of the Prognostic Model
GSE66405	Germany	GPL17077	Wilms tumor (n=28), normal kidney (n=4)	Identification of hub genes
GSE11024	USA	GPL6671	Wilms tumor (n=27), normal kidney(n=12)	Identification of hub genes
GSE73209	Sweden	GPL10558	Wilms tumor (n=32), normal kidney (n=6)	Verification of hub genes
GSE110696	USA	GPL23126	Wilms tumor (n=13), normal kidney (n=6)	Verification of hub genes

### Transcriptomic analysis of differential gene expression

Differential expression analysis was performed using the R package “limma” (version 4.0.3), which employs a negative binomial model to estimate log2 fold changes and p-values for each gene. Genes with a p-value of < 0.05 were considered significantly differentially expressed. Volcano plots were generated to illustrate the distribution of differentially expressed genes (DEGs) between Wilms tumor (WT) and normal kidney tissues, utilizing data from the GEO database. The raw data, downloaded in MINiML format, underwent differential expression analysis via the “limma” package in R software. To reduce false positives, adjusted p-values were calculated using the Benjamini-Hochberg method, with DEGs defined as those with an adjusted p-value < 0.05 and a log2 fold change > 1.3 or < -1.3. The overlapping DEGs from datasets GSE66405 and GSE11024 were compared using a Venn diagram, and the overlap coefficient was calculated to assess consistency between the datasets. Additionally, validation of key hub genes was conducted using two independent datasets, GSE73209 and GSE110696 (5).

### Functional enrichment analysis: gene ontology and KEGG pathways

To characterize the biological functions and pathways associated with differentially expressed genes (DEGs) identified from GEO datasets, functional enrichment analyses were performed using Gene Ontology (GO) and Kyoto Encyclopedia of Genes and Genomes (KEGG) pathways. The “clusterProfiler” package in R was employed for this purpose, with significant pathways defined by an adjusted p-value < 0.05 using the Benjamini-Hochberg correction. Enrichment analyses were conducted across three categories: biological processes (BP), molecular functions (MF), and cellular components (CC), with a minimum of 10 genes per category included in the analysis. Gene sets were retrieved from the Molecular Signatures Database (MSigDB) (6) to ensure comprehensive coverage. Visualization of the enriched GO terms and KEGG pathways was accomplished using the “enrichplot” R pack (7).

### Survival analysis

To evaluate the prognostic significance of key hub genes, survival analysis was performed using the TARGET Wilms tumor (WT) cohort. Patients were stratified into high- and low-expression groups based on the median expression level of each gene. Kaplan-Meier survival curves were generated to assess the relationship between gene expression levels and overall survival (OS) or progression-free survival (PFS). Statistical significance was determined using the log-rank test, with p-values < 0.05 considered significant. Hazard ratios (HR) and 95% confidence intervals (CI) were calculated using univariate Cox proportional hazards regression to quantify the impact of gene expression on patient survival outcomes.

### Development and validation of the prognostic risk model

The prognostic risk model was developed by integrating gene expression data from the TARGET Wilms tumor cohort with patient survival outcomes. Key survival-associated genes were identified using the least absolute shrinkage and selection operator (LASSO) regression, combined with 10-fold cross-validation to optimize the model. Risk scores were subsequently calculated for each patient based on the regression coefficients and corresponding gene expression values, with the calculation method detailed in a previous study.

### Cell culture

HEK 293T cells were acquired from the American Type Culture Collection (ATCC, USA), and WiT49 cells were obtained as previously reported (5). WiT49 cells were cultured in a 1:1 mixture of high-glucose Dulbecco’s Modified Eagle Medium (DMEM) and Nutrient Mixture F-12 (DMEM/F-12), supplemented with 10% fetal bovine serum (FBS), 100 U/mL penicillin, and 100 µg/mL streptomycin, as described in previous studies. HEK 293T cells were maintained in DMEM with 10% FBS, supplemented with the same antibiotics. Both cell lines were routinely tested for mycoplasma contamination and confirmed to be mycoplasma-free before use in experiments, ensuring cell line authenticity and reliability for downstream applications.

### Transfection

Transfection was performed using Lipofectamine 3000 (Invitrogen, USA) according to the manufacturer’s protocol. WiT49 cells were seeded in 6-well plates at a density optimized for 70-80% confluency at the time of transfection. Small interfering RNA (siRNA) targeting GRAMD1A (si-GRAMD1A) and a negative control siRNA (si-NC) were purchased from IGEbio (Guangzhou, China). The specific sequences used for each siRNA are provided in **Table 2**. Transfection efficiency was confirmed 48 hours post-transfection by quantifying GRAMD1A mRNA and protein expression levels using RT-qPCR and Western blot, respectively. This ensured effective knockdown of GRAMD1A, validating the subsequent functional assays.

**Table 2:** Oligonucleotide sequences.

Gene	Target sequence
Negative Control (si-NC)	TTCTCCGAACGTGTCACGT
Si RNA-GRAMD1A-1	CCCACTTATAAGCAGCGTAAT
Si RNA-GRAMD1A-2	CAGACACAAGTAACTCCTCTT

### Western blotting

Total proteins were extracted from WiT49 and HEK 293T cells using RIPA lysis buffer supplemented with protease inhibitors. Protein concentrations were quantified using a BCA protein assay kit (Thermo Fisher Scientific). Equal amounts of protein (25 μg per lane) were subjected to separation by SDS-PAGE and subsequently transferred onto 0.22 μm PVDF membranes (Millipore). The membranes were blocked in 5% non-fat milk diluted in Tris-buffered saline with 0.1% Tween-20 (TBST) for 1 hour at room temperature. Following blocking, membranes were incubated overnight at 4°C with primary antibodies against GRAMD1A (1:1000, NBP1-93730, Novus) or α-Tubulin (1:5000, 11224-1-AP, Proteintech), with α-Tubulin serving as the loading control. After three washes in TBST, membranes were incubated with HRP-conjugated secondary antibodies (1:5000, Thermo Fisher Scientific) for 1 hour at room temperature. Protein bands were visualized using enhanced chemiluminescence (ECL) reagent and imaged with a ChemiDoc XRS+ imaging system (Bio-Rad). The relative expression levels of proteins were quantified using ImageJ software, normalized to α-Tubulin to ensure consistent loading across samples.

### RT-PCR

Total RNA was isolated from Wilms’ tumor (WiT-49) cells and normal renal epithelial (HEK 293T) cells using TRIzol reagent (Invitrogen, USA), following the manufacturer’s protocol. The extracted RNA was quantified using a NanoDrop spectrophotometer (Thermo Fisher Scientific). Complementary DNA (cDNA) was synthesized from 1 μg of total RNA using the M-MLV reverse transcription kit (M1705, Promega). Quantitative PCR (qPCR) was performed on the synthesized cDNA using SYBR Master Mix (DRR041B, TAKARA) with a LightCycler 480 II instrument (Roche). The qPCR cycling conditions included an initial denaturation at 95°C for 5 minutes, followed by 40 cycles of denaturation at 95°C for 10 seconds, annealing at 60°C for 20 seconds, and extension at 72°C for 30 seconds. Relative gene expression levels of GRAMD1A and GAPDH were calculated using the 2^^-ΔΔCt^ method, with GAPDH as the internal control for normalization. Primer sequences used for qRT-PCR are detailed in **Table 3**. All experiments were conducted in triplicate to ensure reproducibility.

**Table 3:** Primers for Quantitative Real-Time PCR (qRT-PCR).

Oligonucleotides	Sequence (5′–3′)
GAPDH-F	GTGGGCAAGGTATCCTG
GAPDH-R	GATTCAGTGTGGTGGGGGAC
GRAMD1A-F	ATAAGCAGCGTAATGAGGACTTC
GRAMD1A-R	AGGGCGCAGGAGTAATCCA

### Cell viability and colony formation assay

WiT49 cells, following transfection with either si-GRAMD1A or si-NC, were seeded into 96-well plates at a density of 3,000 cells per well. Cell viability was assessed at 24, 48, and 72 hours post-transfection using the Cell Counting Kit-8 (CCK-8; Dojindo, Japan). After adding 10 μL of CCK-8 reagent per well, cells were incubated at 37°C in a humidified 5% CO_2_ atmosphere for 2 hours, and absorbance was measured at 450 nm using a microplate reader.

For the colony formation assay, transfected WiT49 cells were plated at a density of 500 cells per well in 6-well plates and cultured for 12 days, with the culture medium refreshed every 4 days. Colonies formed were fixed with 4% formaldehyde for 15 minutes, stained with 0.1% crystal violet, and counted manually under a microscope.

### Transwell assay

Transfected WiT49 cells were resuspended in serum-free DMEM medium and seeded at a density of 5 × 10^4^ cells per insert into the upper chamber of transwell inserts (8 μm pore size; Corning, USA). For the migration assay, inserts without Matrigel coating were used, while for the invasion assay, the membranes were coated with 100 μL of Matrigel (1:8 dilution, BD Bioscience, USA) and allowed to solidify for 1 hour at 37°C. The lower chambers were filled with medium containing 15% FBS, serving as a chemoattractant. After 24 hours of incubation at 37°C in a humidified incubator with 5% CO_2_, non-migratory/invading cells on the upper side of the membrane were carefully removed using a cotton swab. Cells that migrated or invaded to the lower surface were fixed with 4% paraformaldehyde for 10 minutes, stained with 0.1% crystal violet (Solarbio, China) for 30 minutes at room temperature, washed with phosphate-buffered saline (PBS), and counted under a phase-contrast microscope in five randomly selected fields per membrane.

### Statistical analysis

Data analysis and visualization were conducted in R (version 4.0.3). The Kruskal-Wallis test evaluated continuous variable differences, and the Wilcoxon test compared two groups. Hazard ratios (HR) and 95% confidence intervals (CI) were calculated via Cox regression using the survival package. Kaplan-Meier analysis and log-rank tests were used to compare survival curves. Statistical significance was set at p < 0.05 (*p < 0.05, **p < 0.01, ***p < 0.001).

## Result

### Identification of differentially expressed genes in Wilms’ tumors patients

**Figure 2:**
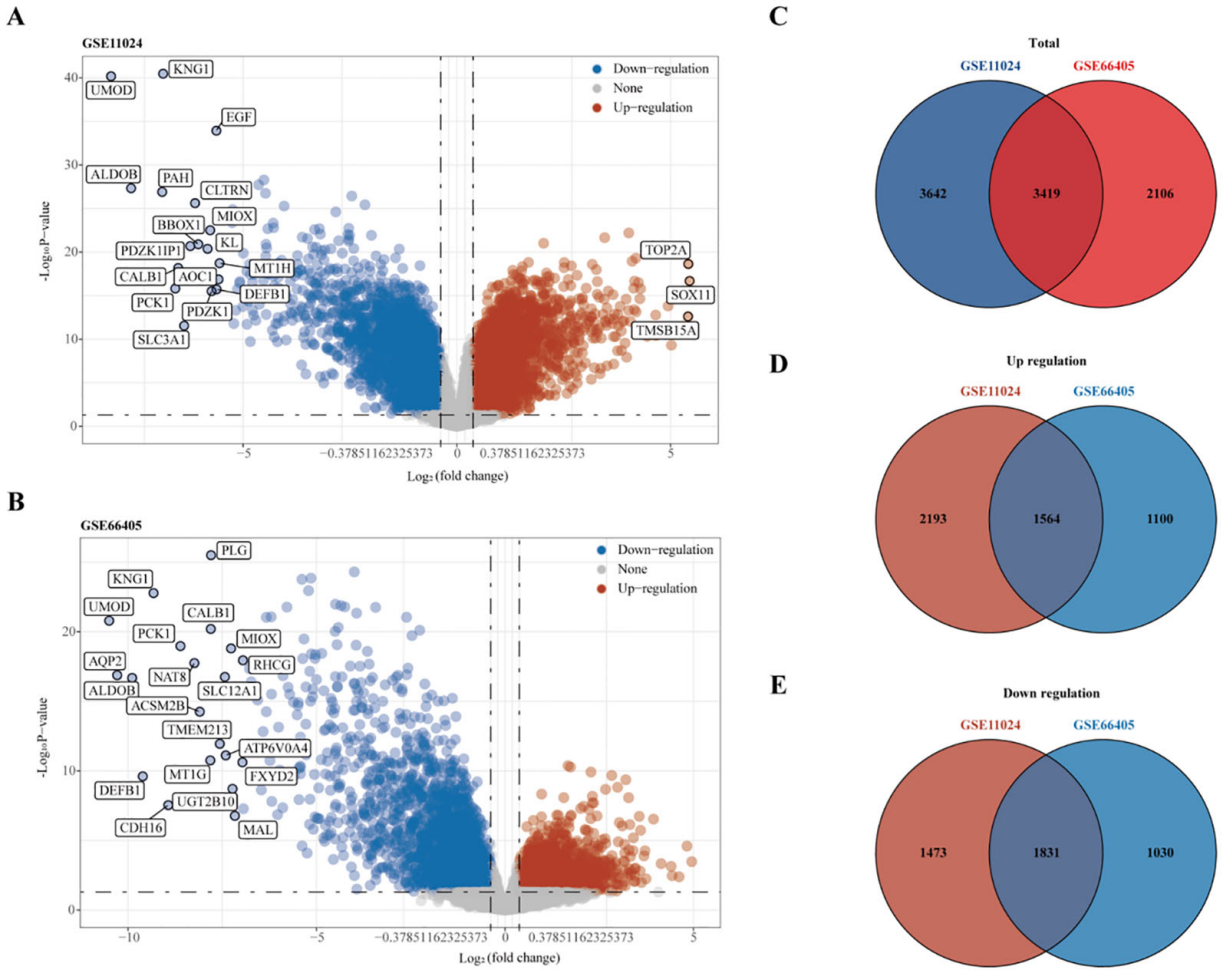
Differential gene expression analysis. **(A)** Volcano plot depicting differentially expressed genes (DEGs) in dataset GSE11024. **(B)** Volcano plot depicting DEGs in dataset GSE66405. **(C–E)** Venn diagrams illustrating intersecting gene sets: **(C)** Total intersecting genes between datasets, **(D)** Overlapping upregulated genes, and **(E)** Overlapping downregulated genes.

RNA sequencing data from the GEO datasets GSE11024 (Wilms tumor, n=27; normal kidney, n=12) and GSE66405 (Wilms tumor, n=28; normal kidney, n=4) were analyzed to determine differentially expressed genes (DEGs). In the GSE11024 dataset, a total of 7,061 DEGs were identified (**Figure 2A**), while 5,525 DEGs were detected in the GSE66405 dataset (**Figure 2B**). To evaluate the consistency of these findings, a Venn diagram was constructed to identify intersecting genes, revealing 3,419 common DEGs between the two datasets (**Figure 2C**). After eliminating duplicates, 3,395 unique DEGs with consistent expression profiles were retained, comprising 1,564 upregulated genes (**Figure 2D**) and 1,831 downregulated genes (**Figure 2E**). These DEGs are likely involved in critical biological processes relevant to the pathogenesis of Wilms tumor.

### Functional enrichment analyses of DEGs

To elucidate the underlying biological roles and potential mechanisms of the identified DEGs, we performed KEGG pathway and Gene Ontology (GO) enrichment analyses separately on the 1,564 upregulated and 1,831 downregulated genes.

**Figure 3:**
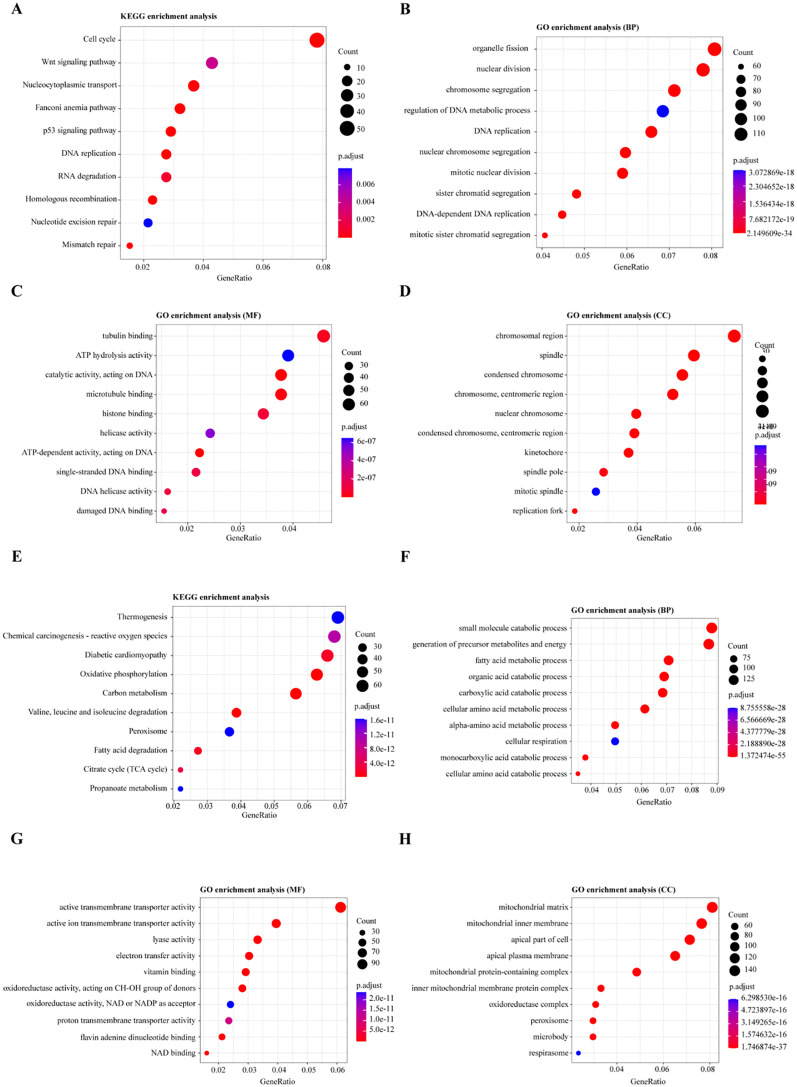
Functional Enrichment Analysis of Differentially Expressed Genes (DEGs). **(A, E)** KEGG pathway enrichment analysis for differentially expressed genes: **(A)** Pathways enriched for upregulated genes, **(E)** Pathways enriched for downregulated genes. **(B, F)** Gene Ontology (GO) enrichment analysis for biological processes involving DEGs: **(B)** Biological processes enriched for upregulated genes, **(F)** Biological processes enriched for downregulated genes. **(C, G)** GO enrichment analysis for molecular functions of DEGs: **(C)** Molecular functions enriched for upregulated genes, **(G)** Molecular functions enriched for downregulated genes. **(D, H)** GO enrichment analysis for cellular components of DEGs: **(D)** Cellular components enriched for upregulated genes, **(H)** Cellular components enriched for downregulated genes.

For the upregulated DEGs, KEGG pathway enrichment analysis identified 18 significantly enriched pathways, with the most notable ones being the Cell cycle, Wnt signaling pathway, and Nucleocytoplasmic transport (**Figure 3A**). GO enrichment analysis revealed 511 significantly enriched Biological Process (BP) terms, primarily associated with organelle fission, nuclear division, and chromosome segregation (**Figure 3B**). A total of 48 enriched terms for Molecular Function (MF) were found, mainly related to tubulin binding, ATP hydrolysis activity, and catalytic activity, acting on DNA (**Figure 3C**). Additionally, 99 enriched terms for Cellular Component (CC) were identified, including chromosomal region, spindle, and condensed chromosome (**Figure 3D**).

For the downregulated DEGs, KEGG pathway enrichment analysis identified 65 significantly enriched pathways, primarily related to Thermogenesis, Chemical carcinogenesis - reactive oxygen species, and Oxidative phosphorylation (**Figure 3E**). GO enrichment analysis of these DEGs identified 670 enriched Biological Process (BP) terms, with key processes involving the small molecule catabolic process, generation of precursor metabolites and energy, and fatty acid metabolic process (**Figure 3F**). For Molecular Function (MF), 168 enriched terms were found, including active transmembrane transporter activity, ion transmembrane transporter activity, and lyase activity (**Figure 3G**). Moreover, 97 enriched Cellular Component (CC) terms were identified, such as mitochondrial matrix, mitochondrial inner membrane, and apical part of the cell (**Figure 3H**). These enrichment analyses suggest that dysregulation of the cell cycle, Wnt signaling, and metabolic reprogramming, particularly involving oxidative phosphorylation and fatty acid metabolism, play critical roles in the progression of Wilms tumor (WT), highlighting potential therapeutic targets.

### Prognostic gene identification and analysis in Wilms tumor patients

**Figure 4:**
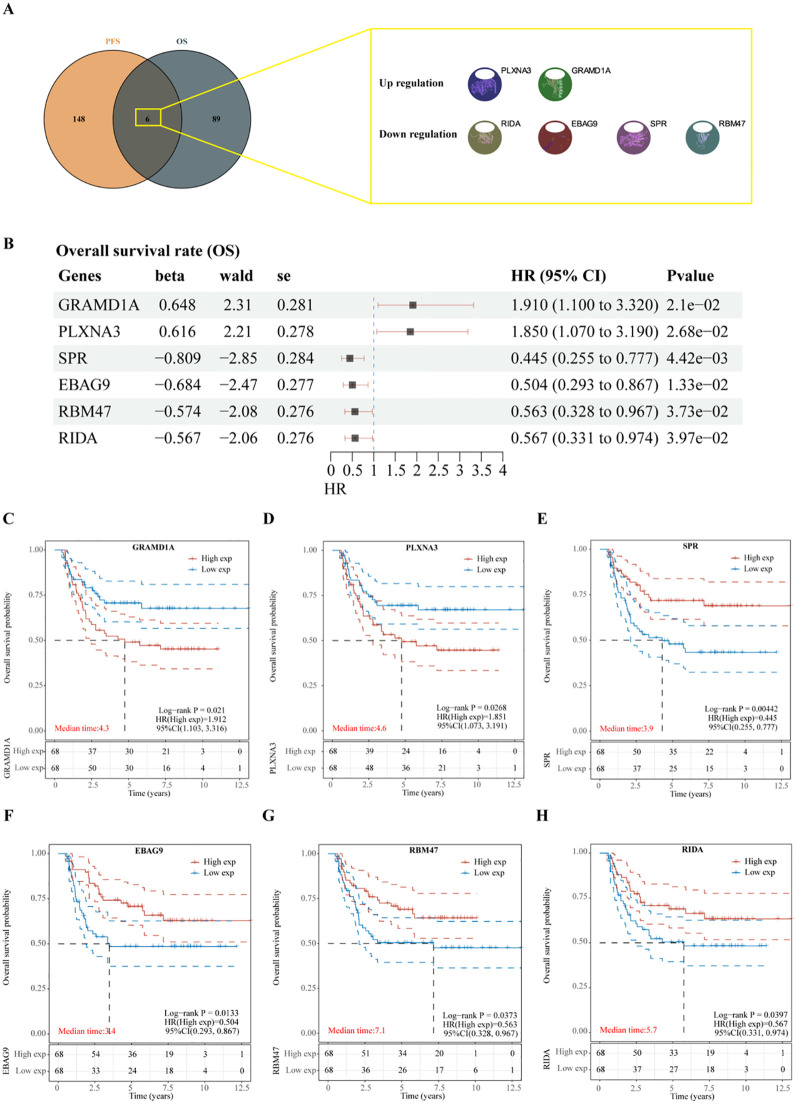
Identification of prognosis-related genes in Wilms tumor. **(A)** Venn diagram illustrating the overlap of genes significantly associated with overall survival (OS) and progression-free survival (PFS). **(B)** Univariate Cox regression analysis of six candidate genes identified from the intersection, assessing their association with overall survival. **(C-H)** Kaplan-Meier survival curves depicting the relationship between the expression levels of each intersecting gene and overall survival in patients with Wilms tumor. High and low expression groups are stratified by median expression levels, demonstrating the prognostic value of each gene.

To identify key prognostic genes in Wilms tumor (WT), clinical data and RNA-seq data from 136 WT patients in the TARGET database were analyzed. A univariate Cox regression analysis was performed to assess the association between the 3,395 DEGs and overall survival (OS) as well as progression-free survival (PFS). As a result, 95 genes were found to be significantly associated with OS (**Table 4**), and 154 genes were significantly associated with PFS (**Table 5**). Further analysis using a Venn diagram revealed six genes that were significantly related to both OS and PFS: GRAMD1A, PLXNA3, SPR, EBAG9, RBM47, and RIDA (**Figure 4A**). Univariate Cox regression analysis was further performed on the six candidate genes identified from the intersection to assess their association with overall survival (**Figure 4B**).

**Table 4:** Univariate analysis of genes associated with overall survival (OS).

Genes	HR	Low 95%CI	High 95%CI	cox p	log rank p
TIPRL	1.756814662	1.203410607	2.564708786	0.003509713	0.000250651
COP1	1.823462434	1.190581968	2.792764663	0.005744986	0.001206594
PIF1	1.495464971	1.013959527	2.205625983	0.042367848	0.001338783
SHISA8	1.248702403	1.060248236	1.470653417	0.007794701	0.001680628
IL17D	1.50148397	1.043117508	2.161265719	0.028734113	0.001822755
SPIN4	1.834456791	1.351923989	2.489216659	9.77E-05	0.001930321
OSR1	1.234454163	1.021795409	1.491372018	0.029000597	0.00412732
NUF2	1.454248167	1.07520195	1.966921407	0.015075066	0.00582547
C1QTNF4	1.376145093	1.107320036	1.710233047	0.003986034	0.0059916
C1orf112	1.630452931	1.111555904	2.391581703	0.012382681	0.005999161
ADGRG2	1.238719322	1.041419129	1.473398668	0.015586939	0.007478738
AGTPBP1	1.45397106	1.031918765	2.048641729	0.032389788	0.00859405
CDCA8	1.452569527	1.026466552	2.055554783	0.035081076	0.009902179
PPP2R3C	1.626659212	1.007458823	2.626430113	0.046550804	0.010140202
PSMA6	1.575850875	1.000785216	2.481357577	0.049605237	0.010287373
AKIRIN2	1.991093122	1.165418291	3.401741548	0.011730875	0.011816117
CENPW	1.443735509	1.102226692	1.891055836	0.007658404	0.012012481
VRK1	2.077018397	1.299090835	3.320788127	0.002266855	0.013275903
GLMN	2.063180306	1.189508416	3.578548012	0.009949171	0.013290057
RPA2	2.000900412	1.097394214	3.648280997	0.023621858	0.013490171
CCNE1	1.571173279	1.189693895	2.074975322	0.001452532	0.014230065
NSMCE4A	1.853558573	1.052900703	3.263061155	0.032467369	0.015047131
PHF19	1.406877144	1.097968257	1.802696285	0.006957695	0.015916613
NUP133	1.501261829	1.016091732	2.218094103	0.041338411	0.016440961
KIF2A	1.390279561	1.03415221	1.869045233	0.029081134	0.016595332
GRAMD1A	1.755503142	1.135142035	2.714894866	0.011413242	0.018996311
ABT1	2.572926199	1.32714036	4.988130438	0.005143757	0.019668082
TACC3	1.58894975	1.092808064	2.31034286	0.015322538	0.020978647
TMEM190	1.205229048	1.030959073	1.408957055	0.019150494	0.022088391
TUBGCP3	1.881905231	1.144265872	3.095056302	0.012743503	0.024042305
THOC6	1.787220388	1.108205422	2.882278547	0.017251085	0.02448834
PLXNA3	1.528282808	1.033812133	2.259258009	0.033445209	0.024876609
CTPS1	1.437874972	1.004773673	2.057661833	0.047032533	0.029099379
DUSP12	1.926419411	1.179968062	3.14507813	0.008750163	0.029576678
C1QTNF7	1.315529626	1.042401071	1.660222964	0.020904051	0.031189164
CAPZA1	2.254189711	1.184848638	4.288624801	0.013255284	0.031337203
MTFR2	1.66938361	1.061910061	2.624366921	0.026404205	0.031973177
GNL2	1.777043536	1.044683589	3.022813571	0.033901265	0.032367575
HSF2BP	1.486275019	1.012677587	2.181359063	0.042937337	0.033325401
CDC20	1.44072634	1.011398867	2.052298509	0.043098787	0.033941001
C1orf174	2.65734443	1.281856716	5.508789969	0.008600365	0.034476318
PFDN4	1.446186213	1.025823359	2.038805751	0.035250762	0.035363107
UTP11	2.70765169	1.329292676	5.515247175	0.006066764	0.036904537
WTAP	1.78356831	1.072838848	2.965138632	0.025676965	0.03799388
DMRT1	1.266783508	1.028076918	1.560914779	0.026425859	0.043010662
DDX20	1.903173741	1.190851581	3.041579946	0.007142288	0.045274171
CDCA4	2.017617197	1.217481286	3.343607169	0.006459317	0.047015168
EXOSC8	2.003960953	1.181432418	3.399144496	0.009925918	0.047502113
LNX1	0.655408471	0.437693917	0.98141703	0.040264572	0.000105594
MAL	0.85658464	0.761558123	0.963468477	0.009871979	0.00055848
CLDN19	0.772363119	0.598705076	0.996391732	0.046835799	0.000678952
PDE8B	0.451433914	0.237869724	0.8567403	0.014976008	0.000711805
IGFBP5	0.808367481	0.682707437	0.957156681	0.013589189	0.001119164
NPNT	0.737134646	0.597864164	0.908847727	0.004310024	0.001252322
SATB2	0.704493278	0.536701612	0.924742479	0.011613819	0.002137404
MECOM	0.812512852	0.679193275	0.972001872	0.023176572	0.002579523
HS6ST2	0.76012737	0.630933232	0.91577617	0.003905944	0.002582468
EMCN	0.687814669	0.516777488	0.915459805	0.010303314	0.002679706
SPR	0.751940677	0.581462703	0.972400772	0.029756857	0.003454253
SMIM24	0.814955346	0.687422934	0.966147889	0.018443974	0.004815277
P3H2	0.7871294	0.640070155	0.967976224	0.023303875	0.006009044
GPR160	0.671136766	0.48370185	0.931202886	0.017008172	0.006650971
EMX1	0.649971266	0.4393624	0.961535732	0.031062068	0.009299515
ALDH1A1	0.793284702	0.665945792	0.944972738	0.00948781	0.009818902
DECR1	0.571393189	0.353658937	0.923178074	0.022223541	0.010331775
EBAG9	0.43410922	0.225814939	0.834536527	0.012335962	0.011685174
KCNJ16	0.771697276	0.618974268	0.962102493	0.021260339	0.012912402
SCD5	0.626903688	0.463235298	0.848398719	0.002486546	0.013507926
SLC6A16	0.589358994	0.401396879	0.865338127	0.006975152	0.015113959
ENPP6	0.594086874	0.356709766	0.989429634	0.045414532	0.015156223
GULP1	0.741487245	0.552994486	0.994229324	0.045647682	0.01533002
SDC1	0.76439825	0.590182258	0.990041088	0.0417699	0.015642824
MTSS1	0.589671376	0.41560547	0.836640413	0.003083837	0.016547087
METTL7A	0.586535233	0.445269518	0.772618754	0.000147727	0.017681268
GPR155	0.593313803	0.420479133	0.837190816	0.002963735	0.017772259
TMEM61	0.718427331	0.536316792	0.962374919	0.026617218	0.018539695
MCEE	0.415169648	0.201483232	0.855484771	0.017167172	0.019459521
SH3YL1	0.572823931	0.334983981	0.979531184	0.041798777	0.019962121
MTUS1	0.661195393	0.474913244	0.920545706	0.014273475	0.021554072
ERBB3	0.753176702	0.607305862	0.93408475	0.009857096	0.024009012
IL1R2	0.479448812	0.237789452	0.966700418	0.039915762	0.024227348
SLC16A4	0.626386753	0.444488564	0.882723192	0.007524781	0.025804304
PRR15	0.75925299	0.594195939	0.97015995	0.027651916	0.02710812
SOST	0.784385382	0.637156873	0.965634138	0.022040551	0.031843763
EVA1A	0.616497395	0.438346976	0.867050667	0.005439009	0.032212615
SOSTDC1	0.758613788	0.634904015	0.906428163	0.002353157	0.03222514
ARHGEF37	0.546600328	0.340472204	0.877522205	0.012387479	0.032366752
AKR1C3	0.810307472	0.66016746	0.994593401	0.044239356	0.032565643
NDRG1	0.702346175	0.515974883	0.956035198	0.024721334	0.033944459
RBM47	0.792166575	0.635756461	0.987057027	0.037889525	0.034975228
RIDA	0.604480658	0.372456941	0.981044588	0.041607844	0.037433652
NEBL	0.75986108	0.609141962	0.947872412	0.014909594	0.044036208
TMEM139	0.735812711	0.561955461	0.963457753	0.025704657	0.044960352
KLF10	0.67257613	0.454863386	0.994493432	0.046852111	0.047653084
SLC26A7	0.767376885	0.61723473	0.954041073	0.017149411	0.049829071

**Table 5:** Univariate analysis of genes associated with progression-free survival (PFS).

Genes	HR	Low 95%CI	High 95%CI	cox p	log rank p
CSNK1E	1.580697095	1.118961503	2.232966282	0.00938611	5.73E-06
ABR	1.387205962	1.096685528	1.754687495	0.006338984	0.00010987
PCNX2	1.337200613	1.071639885	1.668569363	0.010096461	0.000130022
INTS4	1.732722297	1.214427217	2.472216134	0.002435222	0.000204421
ZNF496	1.297736747	1.039176979	1.620629303	0.021507961	0.000209649
KCNAB3	1.34186556	1.075780718	1.673764132	0.009114083	0.000286244
FZD10	1.216074749	1.034285159	1.429816314	0.017883598	0.000348844
KCNG1	1.279847248	1.085156329	1.509468207	0.003382745	0.000461322
EVL	1.453681782	1.06668072	1.981090203	0.017851402	0.000520706
LRFN5	1.145950613	1.018752944	1.289029707	0.023239913	0.000543073
RBFOX2	1.370377809	1.065840813	1.761928533	0.01400115	0.000618783
MAP3K12	1.332198713	1.017230475	1.744691547	0.037151698	0.000741121
KDM4B	1.43460044	1.070818551	1.921967471	0.015584281	0.000784527
ZNF84	15.3691437	2.115274564	111.6689918	0.006926045	0.000858247
CAMK1D	1.284128183	1.038412214	1.58798709	0.021010087	0.00114445
RPA1	1.569181971	1.15473191	2.13238418	0.003984404	0.001359601
PCSK5	1.441834814	1.125243832	1.847499689	0.003817772	0.001450649
NCBP3	1.532511804	1.166997945	2.012507766	0.002134523	0.001530257
RPS6KA5	1.589117452	1.115590833	2.263638426	0.010289835	0.001563962
PIAS3	1.452207108	1.145266973	1.8414095	0.002073046	0.001834804
NFATC4	1.340051921	1.057951358	1.697374022	0.015221036	0.001867838
NTM	1.283563815	1.099831396	1.497989668	0.001538939	0.002039635
EHMT1	1.31585142	1.011201086	1.712285502	0.041065764	0.002110798
TP53	1.434728974	1.152778993	1.785639088	0.001222663	0.002447496
TTC23	1.609052552	1.116112778	2.319702961	0.010816772	0.002555891
DLC1	1.316603209	1.001330476	1.731140767	0.048897504	0.002575198
MEIS1	1.177150339	1.00361604	1.380690288	0.045035581	0.002710883
ELAVL4	1.149155189	1.024503583	1.288973187	0.017634762	0.003848663
LPAR2	1.384223054	1.039655994	1.842987944	0.025997761	0.003854636
VWCE	1.370098124	1.115110637	1.683392487	0.002726956	0.004474024
PLXNA3	1.368154978	1.058538151	1.768333094	0.016641389	0.004959709
HMCN1	1.159910469	1.025230877	1.312282263	0.018490123	0.00515678
GEMIN4	1.464311678	1.098315943	1.952269475	0.009348611	0.005558763
ZNF362	1.342117938	1.042238328	1.728280864	0.022571406	0.005590537
FMNL3	1.314845082	1.006530689	1.717600475	0.044674071	0.006184118
POLR1E	1.442837357	1.007806481	2.065654147	0.045237696	0.006404605
NYNRIN	1.28509393	1.039597136	1.588563832	0.020395881	0.007499206
CRAMP1	1.438203294	1.088301817	1.900602097	0.010622346	0.007513487
GRAMD1A	1.358168328	1.031968775	1.787477732	0.028924468	0.007781393
AFF3	1.19071552	1.007530331	1.407206718	0.040561602	0.00812401
ZBED4	1.271246517	1.00509963	1.607868173	0.045241273	0.008193982
TRIM46	1.338993806	1.082587971	1.65612815	0.007109776	0.008920982
FBN1	1.36260469	1.085830337	1.709927859	0.007567501	0.008950637
ZNF71	1.387424639	1.02343538	1.880868266	0.034929485	0.008960898
NARF	1.397320242	1.027556907	1.900141828	0.032899836	0.009422015
KIF26B	1.164514437	1.026523736	1.321054571	0.017944377	0.009560025
ZCCHC18	1.505967527	1.065977401	2.127566861	0.020213139	0.009600067
SSR2	1.465255737	1.006059604	2.134042919	0.046430739	0.010883315
UIMC1	1.799250306	1.150426742	2.814000705	0.010050699	0.01118087
TTC28	1.336036145	1.070073926	1.668102117	0.010528666	0.011763395
SAMD14	1.525046078	1.181179262	1.969019959	0.001206892	0.011866578
VASH1	1.308480916	1.047480747	1.634514347	0.017854269	0.012350723
FLRT2	1.25678335	1.075461237	1.46867626	0.004038747	0.012900503
C3orf70	1.220203378	1.064928456	1.398118602	0.004159242	0.013137532
ADPRM	1.506907189	1.099215887	2.065808276	0.010843727	0.013395987
MAP4K4	1.287421818	1.022725215	1.620625868	0.031451814	0.013793367
RNF165	1.271138451	1.001922191	1.612693058	0.048179836	0.013822187
NID2	1.239744933	1.034658483	1.485482913	0.01984607	0.016985764
DACT1	1.246276415	1.051011514	1.477819112	0.011334733	0.018545161
TMEM39B	1.579770529	1.077290143	2.316622818	0.019226173	0.01855494
PANX1	1.390614278	1.090596378	1.773165683	0.007828026	0.018700654
MEX3A	1.161326246	1.007985634	1.33799392	0.038447498	0.01884567
DSE	1.283067707	1.041205484	1.581112244	0.019342702	0.021416578
NAV1	1.239611691	1.00798052	1.524471073	0.041822523	0.021968688
KDM5B	1.29154473	1.056963384	1.578188815	0.012360356	0.02209794
FBXL7	1.226156546	1.005588635	1.495104284	0.043900022	0.023238906
ZFP69B	1.254483053	1.003015501	1.568996419	0.04699522	0.023441867
NRP2	1.227409068	1.038103726	1.451235538	0.016506661	0.023708192
TRIO	1.430452885	1.086551966	1.883200732	0.010722258	0.023862816
PLCB4	1.279631565	1.0237379	1.599488446	0.030306814	0.025305896
ANKRD50	1.289585175	1.038911571	1.600742518	0.021104369	0.026474236
SH3PXD2A	1.253898441	1.020232857	1.541080832	0.031528623	0.027481675
RIC1	1.325689181	1.010504876	1.739181913	0.041809812	0.028038738
PHLDA1	1.208956502	1.00609786	1.452717358	0.042884366	0.032274574
AP4B1	1.62439269	1.007351048	2.619396304	0.046590109	0.033543815
BRD1	1.324932004	1.017277143	1.725630844	0.036885926	0.03512009
SLCO5A1	1.270782816	1.071268317	1.507455171	0.005959498	0.036062085
FGD6	1.34503297	1.044750596	1.731622549	0.021471358	0.037295006
KIF3C	1.323868849	1.048390111	1.671733366	0.018425365	0.037583849
STXBP5	1.212392443	1.022362294	1.437744178	0.026813276	0.037663663
GPR161	1.278569537	1.0449542	1.564413121	0.016981215	0.038115864
PHF21A	1.388643399	1.067472751	1.806444698	0.014425298	0.038772577
MYB	1.246138355	1.041142143	1.491497399	0.016411453	0.040344548
MAP4K5	1.531806201	1.068216534	2.196586705	0.020406793	0.040866176
DCHS1	1.291125821	1.055996248	1.578609668	0.012732388	0.042594127
CSPG4	1.226561709	1.011606075	1.487193151	0.037769713	0.044022901
CCDC80	1.344391604	1.082593841	1.66949849	0.00740336	0.045379568
COL6A2	1.201506173	1.016836578	1.419713959	0.031078997	0.045930288
BUD13	1.42202447	1.020106189	1.982297151	0.037762661	0.046264126
TNFRSF25	1.267925365	1.001304387	1.605540485	0.048748339	0.047630083
HIC1	1.294387456	1.093251681	1.532528069	0.00274736	0.047995878
ST3GAL2	1.315736988	1.028935073	1.682481108	0.028717362	0.04902414
RIDA	0.426206125	0.29635584	0.612951177	4.22E-06	6.66E-06
NDUFB9	0.502177474	0.345702441	0.729477681	0.000299494	4.17E-05
EBAG9	0.387721722	0.23600318	0.636975036	0.000183568	0.000106555
GHITM	0.402770179	0.235565909	0.688655749	0.000890581	0.000217676
LACTB2	0.600843672	0.450108244	0.802058445	0.000546907	0.000474919
SPR	0.784084115	0.655275558	0.938212775	0.007895263	0.000476346
MRPL34	0.59359025	0.411291207	0.856690778	0.005331745	0.000547149
DDC	0.721006662	0.544324336	0.955038332	0.022564962	0.000974524
SOWAHA	0.797470665	0.681753071	0.932829624	0.004665994	0.001180363
ATP5PD	0.626492028	0.407119303	0.964071856	0.03347477	0.001479945
NDUFA2	0.714191008	0.516160256	0.988198511	0.042192459	0.00154896
GPT	0.529711045	0.294814958	0.951762398	0.033559294	0.001571039
LRP11	0.684096846	0.532358887	0.87908459	0.00300555	0.001741648
DBI	0.558515428	0.406649393	0.767096888	0.00032118	0.001766336
ITM2B	0.709661353	0.544528524	0.924872094	0.011152424	0.001963291
DDO	0.547064066	0.386503101	0.774325204	0.000666934	0.002154464
TUBA4B	0.168217238	0.04282123	0.660817991	0.010667348	0.002337593
NDUFB2	0.687610706	0.479571695	0.98589739	0.041628072	0.003646638
ATP5F1C	0.539926981	0.331956925	0.878189679	0.01301569	0.004128072
EMC7	0.657122624	0.432867436	0.997557467	0.048672859	0.004693375
ATP6V0B	0.662936975	0.473456531	0.92824875	0.016689381	0.005374602
TPD52	0.728515735	0.543782801	0.976005815	0.03377686	0.005751571
DNAJC19	0.543781058	0.344617345	0.858046884	0.008848912	0.00579223
GINM1	0.635862667	0.43610274	0.927124034	0.018610598	0.005909956
GPX3	0.857607882	0.738806056	0.995513333	0.043479873	0.006453786
PRADC1	0.749024917	0.562264424	0.997819358	0.048281478	0.00648136
NDUFB3	0.597161174	0.392929702	0.907545209	0.015768101	0.007511265
COX5B	0.651523114	0.462069447	0.918654913	0.014527892	0.008208232
GLDC	0.799067854	0.667300518	0.956854397	0.014701421	0.009000779
PCBD1	0.456591549	0.289033347	0.72128647	0.000778224	0.010786434
SNTA1	0.75825679	0.593565099	0.968644149	0.026763295	0.012109923
GPD1	0.620901458	0.424429503	0.90832192	0.014074215	0.012160453
GATM	0.821368399	0.683346905	0.98726729	0.036039071	0.012551424
PLPBP	0.594701461	0.404266543	0.874843178	0.00831699	0.01255462
MORN2	0.538718081	0.332941205	0.871676941	0.011758349	0.013128367
MYOZ1	0.829602257	0.694523644	0.990952448	0.039379629	0.013555841
RBM47	0.823424178	0.711259682	0.953276833	0.009311031	0.014139201
ENPP4	0.708592581	0.531930936	0.94392601	0.018553792	0.014632167
ATP5MC1	0.632959655	0.435150583	0.920688009	0.016748366	0.0151189
COMMD8	0.668314377	0.476117487	0.938096414	0.019842078	0.015256057
PRKAA2	0.626195393	0.468834804	0.836372784	0.001524224	0.015530858
C12orf75	0.807590082	0.659981209	0.988212591	0.037976941	0.016031676
GPD1L	0.633772158	0.440834716	0.911151351	0.013803902	0.017723141
PLPPR1	0.805270497	0.678863968	0.955214304	0.012923026	0.018758749
HSBP1L1	0.833176118	0.695037148	0.998770276	0.048465069	0.020361537
COX14	0.604817477	0.406715479	0.899410519	0.013006046	0.020950613
IDH3A	0.528858854	0.328714174	0.850865919	0.008649441	0.021227271
PNKD	0.693215131	0.498635276	0.963724873	0.029274278	0.021856794
OMA1	0.588966722	0.375973577	0.922622813	0.02079812	0.022128833
TSPAN33	0.81706314	0.695250244	0.960218541	0.014175442	0.023334874
MDH1	0.461306616	0.273833445	0.777128571	0.003642785	0.025177658
CYC1	0.583163029	0.39160827	0.86841659	0.007945472	0.026381862
NDUFA5	0.672082891	0.453608829	0.995781791	0.047587055	0.02920604
LGALS2	0.750325147	0.613120034	0.918234268	0.005305742	0.029206256
TMBIM6	0.642535985	0.420920353	0.980832809	0.040399433	0.034082184
ASS1	0.799211532	0.679538804	0.939959673	0.006766772	0.035180334
TUBA4A	0.811904975	0.678269355	0.971870074	0.023151152	0.037232404
CYCS	0.719193332	0.525827186	0.983667376	0.039109855	0.041070375
PHYH	0.73067417	0.546827534	0.976331127	0.033842262	0.04327263
NAA20	0.632143639	0.424698116	0.940916774	0.023817004	0.045755552
COQ10B	0.655950608	0.451306903	0.953389363	0.027094984	0.047054764
PLAAT3	0.737435305	0.618937917	0.878619348	0.000654835	0.047250051

**Figure 5:**
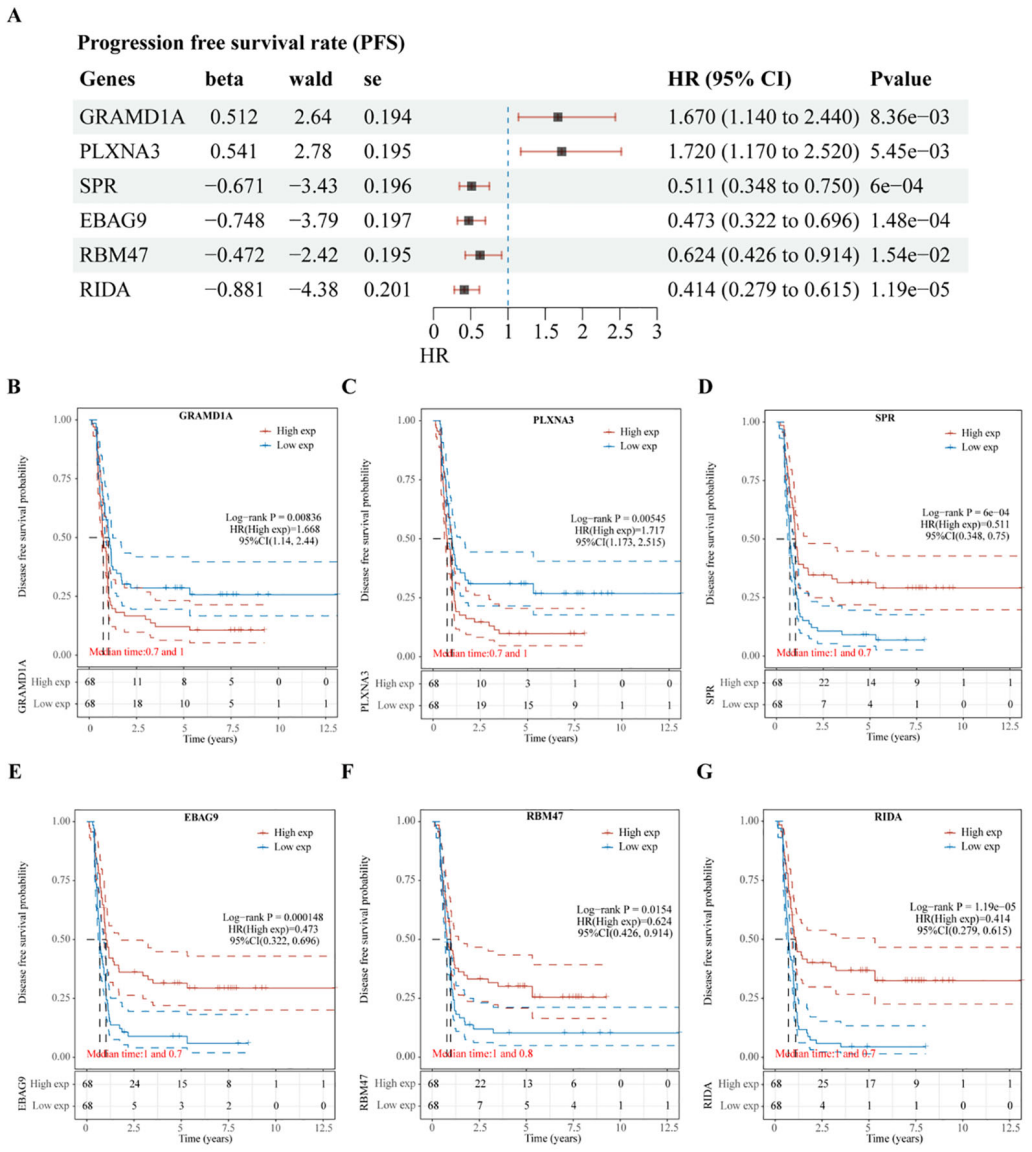
Validation of Prognostic Significance of Six Key Genes in Relation to Progression-Free Survival (PFS). **(A)** Univariate Cox regression analysis of six intersecting genes to determine their prognostic significance for PFS. **(B–G)** Kaplan-Meier survival curves illustrating the association between the expression levels of each of the intersecting genes and PFS in Wilms tumor patients. Each plot shows the impact of high versus low gene expression on patient outcomes, providing insight into their potential prognostic value.

Subsequent analysis of the expression levels of these six genes in relation to OS demonstrated that high expression of GRAMD1A (**Figure 4C**) and PLXNA3 (**Figure 4D**) was associated with poor OS, while low expression of SPR (**Figure 4E**), EBAG9 (**Figure 4F**), RBM47 (**Figure 4G**), and RIDA (**Figure 4H**) was correlated with unfavorable OS outcomes. Similarly, the expression levels of these six genes were analyzed in relation to PFS, as shown in **Figure 5A**. The relationship between the expression of these genes and PFS revealed that high expression of GRAMD1A (**Figure 5B**) and PLXNA3 (**Figure 5C**) was linked to poor PFS, whereas low expression of SPR (**Figure 5D**), EBAG9 (**Figure 5E**), RBM47 (**Figure 5F**), and RIDA (**Figure 5G**) was associated with poor PFS outcomes. These findings suggest that both high expression of GRAMD1A and PLXNA3, and low expression of SPR, EBAG9, RBM47, and RIDA are associated with poorer OS and PFS in WT patients, highlighting their potential as prognostic biomarkers.

### Construction of the prognostic risk model and analysis

**Figure 6:**
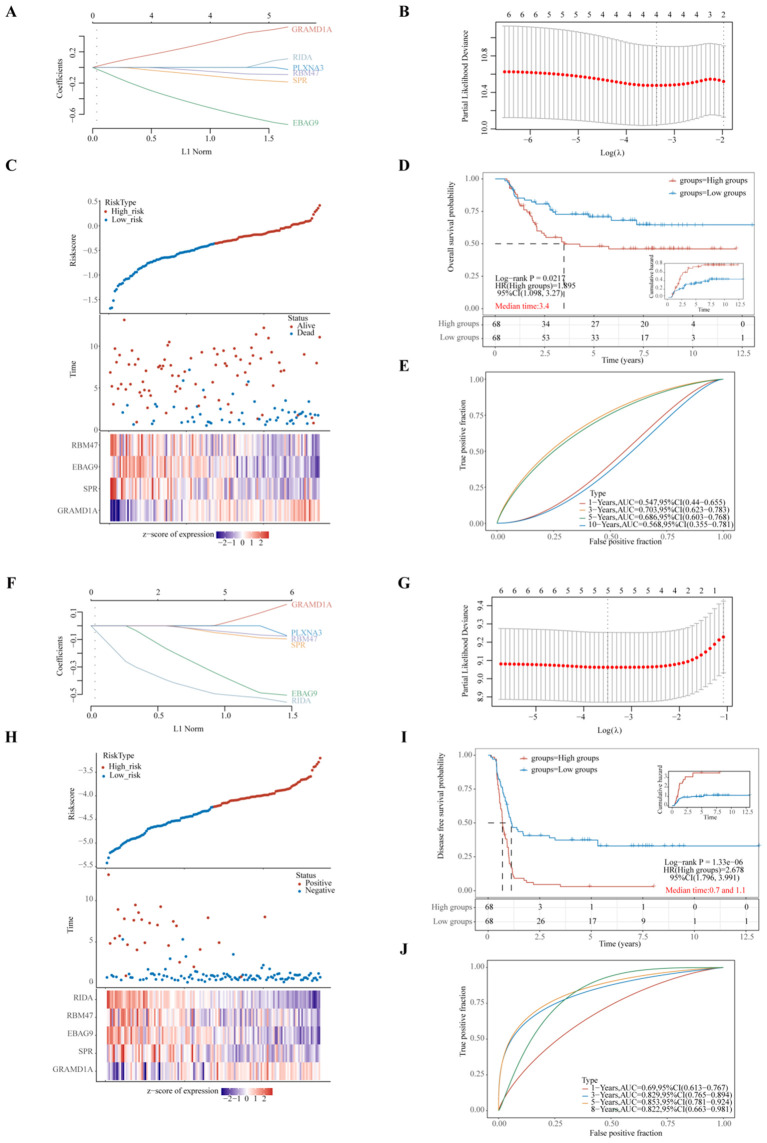
Evaluation of Prognostic Risk Score Models Based on Six Key Prognostic Genes in Wilms Tumor (WT) Patients. **(A, F)** LASSO (Least Absolute Shrinkage and Selection Operator) regression coefficients for the selected prognostic genes, with optimization performed using the lambda parameter to minimize prediction error. **(B, G)** Partial likelihood deviance as a function of log(λ), demonstrating the selection of the optimal penalty parameter in the LASSO model. **(C, H)** Comprehensive representation of risk score distribution, patient survival status, and a heatmap illustrating the expression levels of key genes within the developed risk model, highlighting the distinction between high-risk and low-risk groups. **(D, I)** Kaplan-Meier survival analysis comparing overall survival (OS) or progression-free survival (PFS) between high-risk and low-risk groups, stratified by the median risk score, emphasizing the prognostic value of the risk model. **(E, J)** Time-dependent Receiver Operating Characteristic (ROC) curve analyses assessing the predictive accuracy of the gene signatures over time for OS or PFS, with Area Under the Curve (AUC) values indicating the robustness of the prognostic model. **(A–E)** represent the LASSO regression model for overall survival, whereas **(F–J)** depict the LASSO regression model for progression-free survival. Each sub-panel contributes to validating the model’s efficiency in predicting clinical outcomes for WT patients.

To further evaluate the predictive capacity of the six identified prognostic genes, two LASSO regression-based prognostic risk models (OS/PFS) were constructed to assess their effectiveness in predicting overall survival (OS) and progression-free survival (PFS) in WT patients (**Figure 6**). The LASSO regression analysis for OS identified four genes (GRAMD1A, SPR, EBAG9, and RBM47) that were significantly associated with OS (**Figures 6A–E**). A risk score formula was developed as follows:

Riskscore=(0.3325)*GRAMD1A+(-0.1101)*SPR+(-0.5246)*EBAG9+(-0.053)*RBM47(lambda.min=0.0344). These results suggest that GRAMD1A is a risk factor for OS, whereas SPR, EBAG9, and RBM47 are protective factors. **Figure 6C** shows the distribution of risk scores, survival status, and a heatmap of the four key gene expression levels. WT patients were stratified into high- and low-risk groups based on the median risk score, with the low-risk group showing significantly better OS (p=0.0217, HR=1.895) (**Figure 6D**). Time-dependent ROC analysis was used to assess the model’s accuracy in predicting WT patient survival. The area under the curve (AUC) at 1, 3, 5, and 10 years was 0.547, 0.703, 0.686, and 0.568, respectively (**Figure 6E**), indicating the model’s potential for OS prediction. The LASSO regression analysis for PFS identified five key genes (**Figures 6F–J**). The following equation was used to calculate the PFS risk score:


Riskscore=(0.0657)∗GRAMD1A+(−0.0769)∗SPR+(−0.4489)∗EBAG9+(−0.0582)∗RBM47+(−0.5172)∗RIDA (lambda.min=0.0302)

In the PFS risk model, GRAMD1A was identified as a risk factor, while SPR, EBAG9, RBM47, and RIDA were protective factors. Patients in the low-risk group had significantly better PFS compared to the high-risk group (p < 0.001, HR = 2.678) (**Figure 6I**). The AUC values for PFS prediction at 1, 3, 5, and 8 years were 0.690, 0.829, 0.853, and 0.822, respectively (**Figure 6J**). In summary, the PFS risk model, constructed using GRAMD1A, SPR, EBAG9, RBM47, and RIDA, demonstrates better predictive accuracy than the OS risk model, making it a valuable tool for prognostic assessment in WT patients. The identified prognostic genes, particularly GRAMD1A, SPR, EBAG9, RBM47, and RIDA, offer significant potential as both prognostic biomarkers and therapeutic targets for improving personalized treatment in Wilms tumor patients.

### Validation of prognostic key hub gene expression in Wilms tumor using external datasets

**Figure 7:**
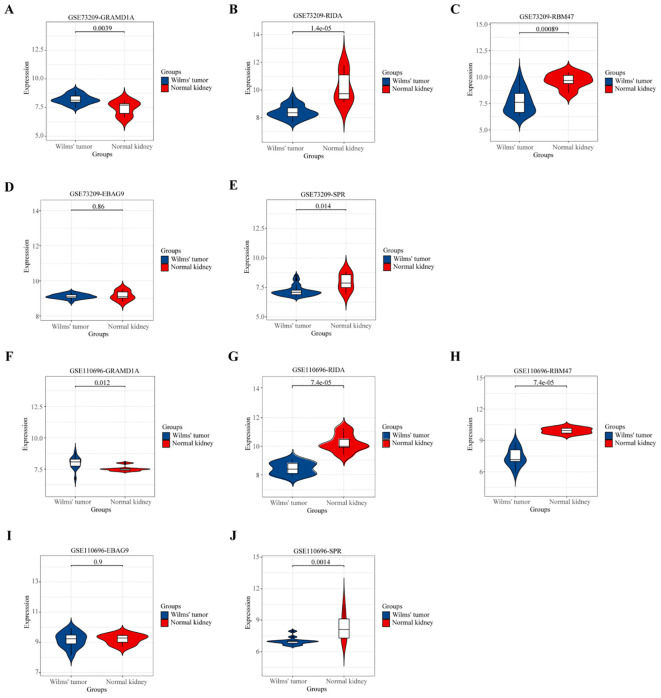
Validation of the Expression Patterns of Five Key Hub Genes in External Datasets. **(A, F)** Validation of GRAMD1A gene expression in Wilms Tumor (WT) samples compared to normal kidney tissues. **(B, G)** Validation of RIDA gene expression levels in WT versus normal tissues. **(C, H)** Validation of RBM47 gene expression to assess differences between WT and normal kidney samples. **(D, I)** EBAG9 gene expression analysis to confirm differential expression in WT. **(E, J)** SPR gene expression comparison between WT and normal tissues. **(A–E)** Validation using the external dataset GSE73209, highlighting expression levels in WT samples versus normal kidney tissues. **(F–J)** Validation using the external dataset GSE110696, demonstrating the robustness and consistency of differential expression for the hub genes identified in our study. These validations confirm the expression trends of the five key hub genes identified, supporting their potential role in Wilms Tumor pathology and their utility as prognostic markers.

To validate the differential expression of the five key genes identified in the prognostic risk model, we utilized two external datasets, GSE73209 and GSE110696, to assess their expression levels in Wilms tumor (WT) and normal kidney tissues (**Figure 7**). In the GSE73209 validation dataset, GRAMD1A (**Figure 7A**) was significantly overexpressed in WT samples compared to normal kidney tissues. Conversely, RIDA (**Figure 7B**), RBM47 (**Figure 7C**), and SPR (**Figure 7E**) exhibited higher expression levels in normal kidney tissues, while EBAG9 (**Figure 7D**) showed no significant differential expression between WT and normal tissues. Consistent findings were obtained from the GSE110696 dataset, further corroborating these expression patterns (**Figures 7F–J**). These results reinforce the robustness and prognostic relevance of the constructed risk prediction model, reinforcing their potential as biomarkers for improving risk stratification and personalized treatment strategies in Wilms tumor patients.

### Cell functional verification

**Figure 8:**
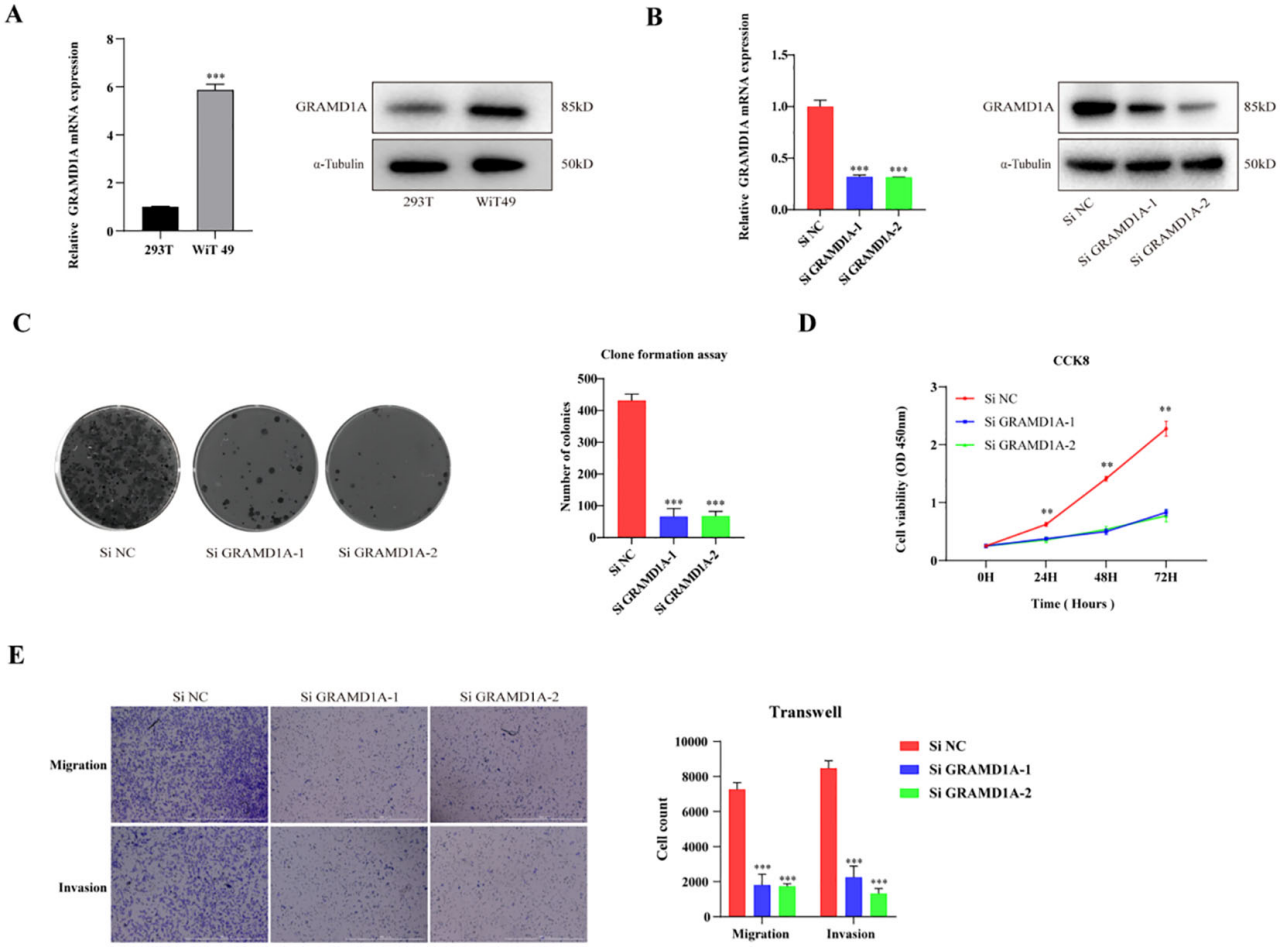
Functional Characterization of GRAMD1A in WiT-49 Wilms Tumor Cells. **(A)** GRAMD1A expression levels in WiT-49 cells, demonstrating baseline expression prior to gene silencing. **(B)** Verification of GRAMD1A knockdown efficiency in WiT-49 cells transfected with GRAMD1A-specific siRNA (siGRAMD1A) or negative control siRNA (siNC). GRAMD1A mRNA levels were measured using RT-qPCR, while protein levels were assessed via Western blot analysis. **(C)** Colony formation assay showing the effects of GRAMD1A silencing on the proliferation capability of WiT-49 cells, highlighting reduced colony formation in GRAMD1A knockdown cells compared to siNC. **(D)** CCK-8 assay results depicting the effect of GRAMD1A knockdown on cell viability over time in WiT-49 cells. Cells transfected with siGRAMD1A exhibited significantly reduced viability compared to control cells, indicating GRAMD1A’s role in promoting cell proliferation. **(E)** Transwell migration and invasion assays used to assess the impact of GRAMD1A silencing on the migratory and invasive behavior of WiT-49 cells. GRAMD1A knockdown led to a marked reduction in both migration and invasion compared to the negative control group. These results collectively demonstrate the role of GRAMD1A in regulating the proliferation, migration, and invasion of WiT-49 cells, further supporting its potential as a therapeutic target in Wilms Tumor. (*p < 0.05, **p < 0.01, ***p < 0.001).

From the four key genes identified in the above analysis, GRAMD1A, which was highly expressed in tumor tissues, was selected for further functional investigation. GRAMD1A expression was confirmed to be upregulated at both the RNA and protein levels in WT cells (**Figure 8A**). To explore the functional role of GRAMD1A, transfection experiments were performed in WiT-49 cells using siRNA. Cells were transfected with either si-NC or si-GRAMD1A, and knockdown efficiency was assessed after 48 hours (**Figure 8B**). Functional assays revealed that silencing GRAMD1A significantly inhibited cell viability, proliferation, migration, and invasion in WiT-49 cells (**Figures 8C–E**).

## Discussion

Wilms tumor (WT) is the most prevalent pediatric renal malignancy (8, 23). Epidemiological studies have revealed variations in WT incidence across different geographic regions and ethnic groups (24), with the disease accounting for approximately 5% of all childhood malignancies in individuals under 15 years of age (25). WT is more common in females, with the incidence peaking in males at 17.9 cases per million person-years at one year of age (26). As an embryonal tumor, WT is thought to arise from developmental arrest or disruption during kidney formation. However, despite significant research efforts, the genetic underpinnings of WT remain incompletely understood, limiting advancements in therapeutic approaches. The identification of prognostic biomarkers and elucidation of the molecular mechanisms driving WT are critical for improving our understanding of this disease and optimizing treatment strategies. In this study, we provide novel insights into the molecular pathogenesis of WT and identify potential prognostic markers that may be instrumental in enhancing patient outcomes. By integrating clinical data and gene expression profiles from multiple datasets, we identified key differentially expressed genes (DEGs) and developed a robust prognostic risk model for WT patients. These findings not only contribute to a deeper understanding of WT biology but also highlight promising molecular targets for therapeutic intervention.

In this study, differential gene expression analysis was performed using transcriptomic data from the GEO database, identifying 3,419 differentially expressed genes (DEGs), including 1,564 upregulated and 1,831 downregulated genes. Subsequently, by integrating data from the TARGET database, six key genes significantly associated with Wilms tumor (WT) prognosis were identified. Among these, GRAMD1A and PLXNA3 were found to be overexpressed in WT and correlated with poorer prognosis, while SPR, EBAG9, RBM47, and RIDA showed reduced expression, which was linked to unfavorable outcomes. These six genes were further used to develop LASSO regression models to predict overall survival (OS) and progression-free survival (PFS) in WT patients. In the OS prediction model, GRAMD1A was identified as a risk factor, whereas SPR, EBAG9, and RBM47 acted as protective factors, with the model achieving an AUC of 0.703 for 3-year OS prediction. In the PFS prediction model, GRAMD1A was similarly identified as a risk factor, while SPR, EBAG9, RBM47, and RIDA were protective factors, resulting in AUC values of 0.690, 0.829, 0.853, and 0.822 at 1, 3, 5, and 8 years, respectively. These results demonstrate the strong prognostic value of these genes for predicting patient outcomes. Notably, the PFS model exhibited superior prognostic value compared to the OS model. Furthermore, our model demonstrated a significant improvement in prognostic accuracy over our previous model (5), as well as those developed by other researchers (27, 28). External validation using two independent datasets confirmed the differential expression of these genes in WT samples compared to normal kidney tissues. Specifically, GRAMD1A was significantly overexpressed in tumor tissues, while RIDA, RBM47, and SPR showed higher expression levels in normal kidney tissues. These findings collectively highlight the prognostic significance of these genes in WT and suggest their potential as targets for future therapeutic interventions.

GRAM Domain Containing 1A (GRAMD1A) mediates non-vesicular cholesterol transport from the plasma membrane (PM) to the endoplasmic reticulum (ER), serving as a key cholesterol transporter (29, 30). Previous studies have demonstrated that GRAMD1A is recruited to autophagosome initiation sites, and its cholesterol transport activity is inhibited by autophagy-related proteins. This inhibition is sufficient to suppress autophagosome biogenesis, indicating that GRAMD1A-mediated cholesterol transport is essential for autophagosome formation (31). In hepatocellular carcinoma (HCC), high GRAMD1A expression is associated with poor prognosis, and it has been shown to promote the self-renewal of liver cancer stem cells and drive hepatocarcinogenesis through the upregulation of STAT5 (32). Although the role of GRAMD1A in tumorigenesis has been largely unexplored in other cancer types, our study is the first to demonstrate that elevated GRAMD1A expression is linked to poor overall survival (OS) and progression-free survival (PFS) in Wilms tumor (WT) patients. Furthermore, GRAMD1A showed substantial diagnostic value in our LASSO-based prognostic model. To elucidate the functional role of GRAMD1A in WT, *in vitro* functional assays were conducted, revealing that GRAMD1A knockdown significantly inhibited tumor cell proliferation, invasion, and migration.

Reactive Intermediate Imine Deaminase A Homolog (RIDA), also referred to as UK114, was first identified in a 1993 study on chromatin-associated proteins, noted for its unusual solubility in perchloric acid. Early structural analyses indicated potential substrate interactions and underscored the conserved nature of this protein family (33). RidA proteins possess conserved enamine/imine deaminase activity, and the absence of RidA in bacteria, plants, and yeast leads to phenotypes such as nutrient deficiencies, mitochondrial maintenance defects, and disruptions in one-carbon (C1) metabolism (34, 35). In oncological research, RIDA/UK114 has been identified as a tumor antigen (36–38). Studies have shown that RIDA/UK114 expression is tissue-specific, with the highest levels found in the liver and kidneys. In hepatocellular carcinoma (HCC), UK114 expression is significantly downregulated at both the mRNA and protein levels, and its decreased expression correlates with the Edmondson-Steiner grade of tumor differentiation, making it a potential biomarker for HCC staging (39). Despite these insights, the precise biological functions of RIDA remain incompletely understood. In this study, we demonstrated that RIDA expression is significantly downregulated in Wilms tumor (WT) compared to normal kidney tissues. Patients with lower RIDA expression had poorer prognoses, and in our prognostic model, higher RIDA expression was identified as a protective factor in WT.

RNA Binding Motif Protein 47 (RBM47) is a single-stranded RNA-binding protein that plays critical roles in various RNA processes, including alternative splicing, RNA stability, and RNA editing (40–42). RBM47 is essential for embryonic endoderm development (43), and conditional expression of RBM47 alleles has been shown to cause fetal intestinal developmental defects and growth retardation (40, 44). As a novel and evolutionarily conserved RNA-binding protein (RBP) in vertebrates, RBM47 is increasingly recognized for its role as a tumor regulator (45). It has been reported to be downregulated in papillary thyroid carcinoma (PTC) tissues and cells, and its overexpression can induce autophagy and suppress PTC cell proliferation (46). In hepatocellular carcinoma (HCC), RBM47 upregulation significantly inhibits tumor progression *in vitro*, primarily through the upregulation of UPF1, serving as a tumor suppressor by acting as a DNA/RNA binding protein at both transcriptional and post-transcriptional levels (47). Furthermore, RBM47 has been shown to modulate intestinal injury and tumorigenesis by altering pathways related to cell proliferation, oxidative stress, and inflammation (48).

Sepiapterin reductase (SPR) is an aldo-keto reductase that catalyzes the NADPH-dependent reduction of pterin derivatives, playing a crucial role in the biosynthesis of tetrahydrobiopterin (BH4) (49). SPR is widely distributed across various tissues and is implicated in numerous diseases, including neurological dysfunction, chronic pain, cardiovascular diseases, and cancer (49). In hepatocellular carcinoma (HCC), elevated SPR expression is significantly associated with shorter patient survival, suggesting that SPR could serve as a potential prognostic marker for HCC. Moreover, SPR is involved in the progression of HCC through a non-enzymatic mechanism, controlling tumor development via the FoxO3a/Bim signaling pathway (50).

The enrichment analysis of upregulated and downregulated genes in Wilms tumor was performed using Gene Ontology (GO) and Kyoto Encyclopedia of Genes and Genomes (KEGG) pathways. Upregulated DEGs were significantly enriched in the cell cycle, Wnt signaling pathway, and nucleocytoplasmic transport pathways, while GO analysis indicated involvement in organelle fission, nuclear division, and chromosome segregation. These results are consistent with the classification of WT as an embryonal malignancy resulting from developmental disruption, highlighting its connection to chromosomal segregation and cell proliferation (51, 52). In contrast, the downregulated DEGs were associated with pathways related to thermogenesis, chemical carcinogenesis, and oxidative phosphorylation. GO analysis further indicated key processes involved in small molecule catabolism, precursor metabolite generation, and fatty acid metabolism, suggesting their role in tumor cell energy metabolism. Previous studies have demonstrated that WT1 suppresses thermogenesis-related genes and modulates metabolic processes (53). Tumor cells often undergo metabolic reprogramming, with oxidative phosphorylation serving as a critical energy source. Its inhibition is emerging as a potential therapeutic target (54). Additionally, oxidative phosphorylation, mediated by macrophages and monocytes, has been closely associated with WT prognosis and clinical outcomes (55, 56).

Although the findings of this study provide valuable insights, several limitations must be addressed. First, the relatively small sample size derived from publicly available datasets, such as TARGET and GEO, may not adequately capture the genetic and clinical heterogeneity of Wilms tumor, potentially affecting the generalizability of our results. The retrospective nature of the study further introduces limitations, including possible biases related to the imbalance of clinicopathological features and treatment heterogeneity across patients. These factors limit the broader application of the findings, especially in diverse clinical settings. Future studies should focus on expanding the cohort size and incorporating more diverse populations to ensure the robustness of the results, while also utilizing prospective study designs to minimize bias. Moreover, although our *in vitro* assays successfully demonstrated the role of GRAMD1A in WT, additional *in vivo* studies are necessary to comprehensively evaluate its therapeutic potential and safety in a physiological context. The molecular mechanisms by which GRAMD1A contributes to tumor progression are not fully elucidated, underscoring the need for further mechanistic exploration. Additionally, while this study identified other prognostic genes (PLXNA3, SPR, EBAG9, RBM47, and RIDA) that could be of clinical significance, their precise roles in WT remain largely unexplored. Detailed mechanistic studies on these genes are warranted to better understand their contribution to WT pathogenesis and their interplay with known oncogenic pathways. Expanding this understanding will help validate these genes as potential biomarkers or therapeutic targets, ultimately enhancing personalized treatment approaches for Wilms tumor.

## Conclusions

In summary, this study identified critical prognostic genes and molecular pathways associated with the progression of Wilms tumor, with GRAMD1A emerging as a key regulator. The prognostic risk models developed in this study provide valuable tools for enhancing the accuracy of outcome prediction and informing personalized therapeutic strategies for patients with Wilms tumor. Continued validation and investigation of these findings in clinical contexts may facilitate the development of novel targeted therapies and contribute to improved clinical outcomes for affected patients.

## Data Availability

The original contributions presented in the study are included in the article/supplementary material. Further inquiries can be directed to the corresponding authors.
